# Extended screening for infectious diseases among newly-arrived asylum seekers from Africa and Asia, Verona province, Italy, April 2014 to June 2015

**DOI:** 10.2807/1560-7917.ES.2018.23.16.17-00527

**Published:** 2018-04-19

**Authors:** Dora Buonfrate, Federico Gobbi, Valentina Marchese, Chiara Postiglione, Geraldo Badona Monteiro, Giovanni Giorli, Giuseppina Napoletano, Zeno Bisoffi

**Affiliations:** 1 Centre for Tropical Diseases (CTD), Sacro Cuore Hospital, Negrar, Verona, Italy; 2 University Department of Infectious and Tropical Diseases, University of Brescia, Brescia, Italy; 3 Prevention Department, Unità Locale Socio Sanitaria (ULSS) 9, Verona, Italy

**Keywords:** screening, asylum seekers, migrants, infectious diseases, parasitic diseases, helminths

## Abstract

Management of health issues presented by newly-arrived migrants is often limited to communicable diseases even though other health issues may be more prevalent. We report the results of infectious disease screening proposed to 462 recently-arrived asylum seekers over 14 years of age in Verona province between April 2014 and June 2015. **Methods:** Screening for latent tuberculosis (TB) was performed via tuberculin skin test (TST) and/or QuantiFERON-TB Gold in-tube assay and/or chest X-ray. An ELISA was used to screen for syphilis. Stool microscopy was used to screen for helminthic infections, and serology was also used for strongyloidiasis and schistosomiasis. Screening for the latter also included urine filtration and microscopy. **Results:** Most individuals came from sub-Saharan Africa (77.5%), with others coming from Asia (21.0%) and North Africa (1.5%). The prevalence of viral diseases/markers of human immunodeficiency virus (HIV) infection was 1.3%, HCV infection was 0.85% and hepatitis B virus surface antigen was 11.6%. Serological tests for syphilis were positive in 3.7% of individuals. Of 125 individuals screened for TB via the TST, 44.8% were positive and of 118 screened via the assay, 44.0% were positive. Of 458 individuals tested for strongyloidiasis, 91 (19.9%) were positive, and 76 of 358 (21.2%) individuals from sub-Saharan Africa were positive for schistosomiasis. **Conclusions:** The screening of viral diseases is questionable because of low prevalence and/or long-term, expensive treatments. For opposing reasons, helminthic infections are probably worth to be targeted by screening strategies in asylum seekers of selected countries of origin.

## Introduction

In 2015, there were an estimated 244 million international migrants, representing 3% of the global population [1]. In recent years, the flow of migrants from Northern Africa to the coasts of southern Italy via the Central Mediterranean migration route has been constantly increasing, reaching 181,126 migrants in 2016 [2]. The term migrant encompasses a heterogeneous population that includes refugees, asylum seekers and economic migrants that come from countries with large differences in the prevalence of diseases [3]. Well-defined screening protocols specifically addressing migrants from different geographical areas are important to detecting some infectious diseases, regardless of whether or not they are causing symptoms. However, the main aim of European health authorities is avoiding the possible spreading of infectious diseases to local populations [4]. Migrants are therefore mostly screened for active [5] and latent tuberculosis (TB) [6,7], human immunodeficiency virus (HIV) infection and chronic viral hepatitis [8,9]. Almost no action aside from local initiatives is taken towards other infections such as parasitic diseases although these often have a higher prevalence than the aforementioned infections [10-14]. Moreover, their treatment is exceedingly shorter and cheaper than treatment for HIV and viral hepatitis, and can prevent severe and even fatal complications in the affected individuals [12,15-17].

In general, the inclusion of infections in a screening programme should take different issues into account. Cost-effectiveness is one of them, but other perspectives should also be considered, such as the heterogeneity of migrant populations in terms of prevalence of specific health problems. Studies have been performed on migrants attending clinics for any reason [13,18], but data from these are not reliable enough to estimate the real prevalence in the general migrant population.

The aim of this study is to estimate the prevalence of a series of infectious diseases, communicable and non-communicable, in a cohort of asylum seekers that recently arrived in Europe and temporarily residing in a series of refugee shelters in Verona province, northern Italy.

## Methods

### Study population and setting

This retrospective observational study includes reporting data from infectious disease screening activities systematically carried out from April 2014 to June 2015 in 14 refugee shelters in Verona province, northern Italy. The shelters are managed by different cooperatives that receive financial support from the Italian government. The study population included asylum seekers over 14 years of age arriving in the last 6 months. Two infectious diseases physicians were in charge of the screening activities and regularly went to the shelters to check on the health of newly-arrived asylum seekers. Those requiring specific workup/treatment were referred either to the local health unit, Azienda Sanitaria Locale (ASL), or to a hospital depending on the level of diagnostic workup/treatment required. Extended screening for infectious diseases was offered to all asylum seekers referred by the physicians to the Centre for Tropical Diseases (CTD) in Negrar, Verona, for the blood sampling. Those who accepted were asked to sign an informed consent form, with parents or a legal guardian signing for individuals less than 18 years of age. Demographic data were registered according to the documents issued by the prefectures where the individuals applied for asylum.

### Screening strategy

In addition to the full blood cell count (FBC), some diagnostic tests for specific infections were proposed:

#### Viral diseases/infections

Screening for HIV infection was performed with an ELISA (Beckman Coulter, Inc.), and a Western blot (Fujirebio Diagnostics) was used as confirmatory test. Depending on the ASL of reference, some individuals were tested for antibodies against hepatitis B virus (HBV), namely the HBV core antibody (anti-HBc) and the HBV surface antibody (anti-HBs) respectively, although most individuals were tested for HBV surface antigen (HBsAg). All assays for HBV were ELISA (Beckmann Coulter, Inc.). Antibodies against hepatitis C virus were also detected with ELISA (Beckman Coulter, Inc.).

#### Bacterial diseases

Screening for syphilis was conducted by screening for *Treponema pallidum* using an IgG ELISA test (Fujirebio Diagnostics) and a rapid plasma reagin, while the *Treponema pallidum* haemaglutination assay (TPHA) was used for confirmatory tests. Screening for TB was conducted with tuberculin skin test (TST), and/or QuantiFERON-TB Gold in-tube assay (QFT-GIT), and/or chest X-ray, depending on the ASL.

#### Helminthic infections

Helminthic infections were screened with the following methods: stool examination for ova and parasites (O and P) after formol-ether concentration with three samples being collected on different days, urine examination after micropore filtration for *Schistosoma haematobium* (only for asylum seekers from sub-Saharan Africa), serology for *Schistosoma* spp. (Schistosoma mansoni ELISA kit, Bordier Affinity Products SA, Crissier, Switzerland) and *Strongyloides stercoralis* (in-house immunofluorescence test, IFAT).

All individuals positive for any screening test were referred to the CTD for the appropriate clinical management. The results were then entered anonymously in an Excel database.

#### Statistical analysis

Descriptive statistics were used to describe the characteristics of the entire cohort. Categorical variables were reported as frequencies and proportions, while quantitative variables were presented as medians with interquartile ranges (IQR). We also investigated associations between age, infections and eosinophilia through Student’s t-test and univariate logistic models. Lastly, we fitted a multivariate logistic regression model to assess a possible association of age and of parasitic infections with the probability of eosinophilia. Statistical analyses were performed using Epi Info programme version 3.5 [19] and R version 3.3.3 [20].

#### Ethical clearance

The study protocol received ethical clearance from the ethics committee for clinical trials in the provinces of Verona and Rovigo (Comitato Etico per la sperimentazione Clinica delle Province di Verona e Rovigo) on 10 May 2017 (protocol number 24126).

## Results

The screening was offered to 481 asylum seekers but as 19 refused, 462 individuals were screened and included in this analysis (Figure). The median age was 24 years (IQR: 20–28) and 95.7% (n = 442) were male. Most came from sub-Saharan Africa (77.5%; n = 358), with others originating from Asia (21%; n = 97) or from North Africa (1.5%; n = 7) (Table 1). Of note, 22.3% of individuals came from Mali.

**Figure:**
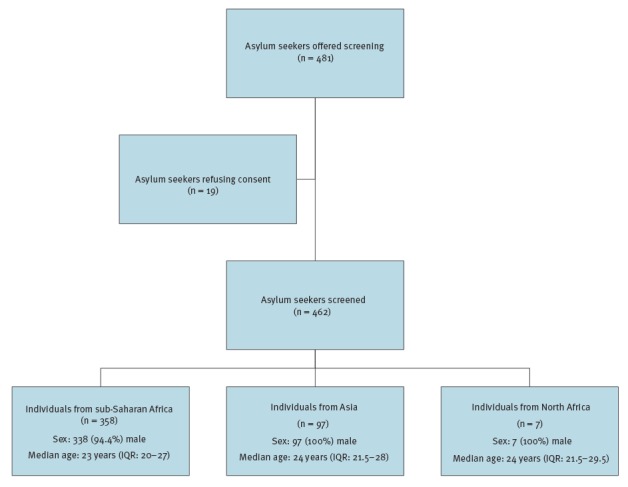
Flow of extended screening for infectious diseases among asylum seekers from Africa and Asia, Verona province, Italy, April 2014–June 2015

**Table 1:** Regions and countries of origin of asylum seekers, Verona province, Italy, April 2014–June 2015

Region	Country	Number of asylum seekers (n)	Percentage within region (%; 95% CI)
**Sub-Saharan Africa**	Angola	1	0.3 (0.1–1.6)
	Burkina Faso	8	2.2 (1.1–4.3)
	Cameroon	3	0.8 (0.3–2.4)
	Congo	1	0.3 (0.1–1.6)
	Côte d’Ivoire	24	6.7 (4.5–9.8)
	Eritrea	6	1.7 (0.7–3.6)
	The Gambia	40	11.2 (8.3–14.8)
	Ghana	39	10.9 (8.0–14.5)
	Guinea	4	1.1 (0.4–2.8)
	Guinea-Bissau	4	1.1 (0.4–2.8)
	Mali	103	28.8 (24.3–33.7)
	Nigeria	81	22.6 (18.6–27.2)
	Senegal	28	7.8 (5.5–11.0)
	Somalia	14	3.9 (2.3–6.5)
	Sudan	2	0.6 (0.2–2.0)
	**Total**	**358**	**100.0**
**Asia**	Afghanistan	1	1.0 (0.2–5.6)
	Bahrain	1	1.0 (0.2–5.6)
	Bangladesh	38	39.2 (30.1–49.1)
	Nepal	1	1.0 (0.2–5.6)
	Pakistan	54	55.7 (45.8–65.2)
	Palestine	1	1.0 (0.2–5.6)
	Sri Lanka	1	1.0 (0.2–5.6)
	**Total**	**97**	**100.0**
**North Africa**	Morocco	6	85.7 (48.7–97.4)
	Tunisia	1	14.3 (2.6–51.3)
	**Total**	**7**	**100.0**

CI: confidence interval.

In the whole cohort, the median eosinophil count was 210/µL (IQR: 120–420), and 144 individuals (31.2%) presented a eosinophil count ≥ 350/µL (i.e. had eosinophilia). In relation to the regions, eosinophilia was present in 105 of 358 (29.3%) individuals from sub-Saharan Africa (median eosinophil count: 550/µL; IQR: 450–740), 38 of 97 (39.2%) individuals from Asia (median eosinophil count: 605/µL; IQR: 465–930), and one of seven individuals from North Africa (eosinophil count: 580/µL). In the Asia subgroup, eosinophilia was present in 18 of 38 individuals from Bangladesh and 18 of 54 individuals from Pakistan.

### Viral diseases/infections

Six of 455 individuals (1.3%) screened were positive for HIV infection. They were all from sub-Saharan Africa: two from Côte d’Ivoire, two from The Gambia, one from Mali and one from Guinea-Bissau. The percentage of HIV-positive individuals of all of individuals screened from sub-Saharan Africa was 1.7% (6/353). Of the 457 people screened for HBV infection by being tested for HBsAg, 53 (11.6%) tested positive. Most (n = 49) came from sub-Saharan Africa, representing 13.8% of people from that region who underwent this screening test (n = 355). The remaining four individuals came from Asia, representing 4.2% of people from that region who were screened (n = 95). In addition, 99 of 338 individuals (29.3%) screened for anti-HBs were positive while 107 of 172 individuals (62.2%) screened for anti-HBc were positive. Of the 118 individuals tested for HCV infection, only one person (0.85%) tested positive.

### Bacterial diseases

In terms of screening for syphilis, 4.5% of individuals from sub-Saharan Africa were positive (16/352), and 1.0% of individuals from Asia were positive (1/95), whereas none of the seven individuals from North Africa were positive. The results of the screening tests for TB are summarised in Table 2. In addition to the data reported in the table, an additional 42 individuals were tested with both TST and QFT-GIT: 16 were negative to both methods, 16 were positive to both, three were positive to TST only and seven were positive to QFT-GIT only. Two-hundred and sixty individuals underwent a chest X-ray, which was normal in 217 (83.5%) cases. Ninety-four people were screened for latent TB exclusively with chest X-ray, and eight (8.5%) of them presented any pulmonary abnormalities. Forty-nine individuals with a positive TST underwent a chest X-ray, and nine presented abnormal pulmonary findings, whereas 30 individuals with a positive QFT-GIT underwent a chest X-ray and seven had pulmonary abnormalities.

**Table 2:** Results of screening tests for latent tuberculosis, Verona province, Italy, April 2014–June 2015

Screening test	Total^a^			sub-Saharan Africa		Asia		North Africa	
	Tested individuals	Positive individuals		Positive/tested individuals		Positive/tested individuals		Positive/tested individuals	
	N	n	%	n/N	%	n/N	%	n/N	%
**TST**	125	56	44.8	37/82	45.1	16/38	42.1	3/5	60
**QFT-GIT**	118	52	44.0	45/94	47.9	7/24	29.2	0/0	0
**Chest X-ray^b^**	260	35	14.5	27/193	13.9	7/61	11.5	1/6	16.7

QFT-GIT: QuantiFERON-TB Gold in-tube assay; TST: tuberculin skin test.

**^a ^**In addition to the data reported in the table, an additional 42 individuals were tested with both TST and QFT-GIT: 16 were negative to both methods, 16 were positive to both, 3 were positive to TST only and 7 were positive to QFT-GIT only.

**^b^** Positive chest X-rays were considered those with any pulmonary abnormalities, including calcified nodules. Abnormalities of other anatomical sites, e.g. heart, vessels, were not included in this analysis.

### Helminthic infections

A stool sample was provided by 270 of 358 (75.4%) individuals coming from sub-Saharan Africa and 79 of 97 (81.4%) individuals coming from Asia; thus, no significant association was observed between region and will to provide a stool sample (chi-squared test, p value = 0.285). Among the seven individuals coming from North Africa, three did not supply a stool sample.

Table 3 shows the main results of stool examination, in relation to region of origin of the asylum seekers. In addition to the data reported in the table, urine was examined for O and P in 96 of the individuals from sub-Saharan Africa, and 20 (20.8%) presented *S. haematobium* eggs. Of the four individuals from North Africa that supplied stool samples, all were negative for O and P. Screening with *S. stercoralis* serology was done for 458 individuals, 91 (19.9%) of whom were positive. Of the latter, 14 (32.6%) had positive stool microscopy for *S. stercoralis* larvae. One additional individual had positive microscopy and negative serology. Hence, 92 of 458 (20.1%) individuals tested were positive to any test for *S. stercoralis*, and 15 (3.3%) had larvae in stool.

**Table 3:** Results of stool examination for ova and parasites^a^, Verona province, Italy, April 2014–June 2015

Region	Individuals screened by stool microscopy	Positive for *Strongyloides stercoralis* larvae		Positive for *Schistosoma mansoni* eggs		Positive for hookworm^b^ eggs		Positive for Ascaris lumbricoides eggs		Positive for *Trichuris trichiura* eggs		Positive for other parasites^a^	
	n	n	%	n	%	n	%	n	%	n	%	n	%
**Sub-Saharan Africa (n = 358)**	270	9	3.3	19	7.0	34	12.6	2	0.7	4	1.5	80	29.6
**Asia (n = 97)**	79	6	7.6	0	0	13	16.5	2	2.5	9	11.4	17	21.5

^a^ Reporting is focused on helminths determined to be clinically relevant, such as soil-transmitted helminths. Some other parasites, both helminths and protozoa, might not have a clinical relevance and are included in the other parasites group (e.g. *Hymenolepis nana* and *Endolimax nana*).

^b^ Includes *Ancylostoma duodenale* and *Necator americanus*.

In terms of regions, 64 of 358 (17.9%) people from sub-Saharan Africa were positive to any test for *S. stercoralis* (serology and/or stool microscopy), whereas 28 of 97 (28.9%) individuals from Asia were. Of 358 individuals from sub-Saharan Africa tested, 76 (21.2%) were positive for at least one test for *Schistosoma* spp. (urine microscopy or stool microscopy or serology). Three of them had negative serology, whereas 32 individuals had positive serology and negative detection of *Schistosoma* spp. eggs.

Table 4 displays a summary of the univariate and multivariate analyses regarding the association between eosinophilia and either age or the main helminthiasis in the entire cohort of asylum seekers, as well as in the sub-Saharan African and Asian subgroups. As expected, people with eosinophilia were more likely to be infected with *S. stercoralis*, *Schistosoma* spp. and hookworm (*Ancylostoma duodenale* and *Necator americanus*). Adjusted ORs highlight a strong association between helminthic infections and the probability of presenting eosinophilia, both in the entire cohort and in individuals from sub-Saharan Africa. In contrast, the presence of *S. stercoralis* was not associated with eosinophilia in individuals from Asia (adjusted odds ratio (OR): 0.76, 95% confidence interval (CI): 0.25–2.13).

**Table 4:** Crude and adjusted odds ratios for the association between helminthic infections^a^ and eosinophilia among all migrants and stratified by region of origin, Verona province, Italy, April 2014–June 2015

Covariate	Eosinophilia present		Eosinophilia absent		Crude OR (95% CI)	Adjusted OR (95% CI)^b^
	n/N	%	n/N	%		
**Total (n = 461)^c,d^**						
Age (median, IQR)	23	(20–26)	24	(20–28)	0.96 (0.93–0.99)	0.98 (0.94–1.02)
*Strongyloides stercoralis*	42/144	29.2	51/316	16.1	2.14 (1.33–3.41)	1.98 (1.11–3.52)
*Schistosoma* spp.	46/144	31.9	36/308	11.7	3.55 (2.17–5.84)	5.13 (2.93–9.18)
Hookworm^e^	32/126	25.4	15/227	6.7	4.81 (2.53–9.53)	5.28 (2.63–11.00)
**Sub-Saharan Africa (n = 357)^d^**						
Age	22	(20–26)	24	(20–28)	0.96 (0.92–1.01)	0.97 (0.91–1.01)
*S. stercoralis*	30/105	28.6	34/251	13.6	2.55 (1.45–4.45)	2.80 (1.37–5.79)
*Schistosoma* spp.	46/105	43.8	36/252	14.3	4.68 (2.78–7.93)	6.38 (3.46–12.05)
Hookworm^e^	22/94	23.4	12/176	6.8	4.17 (1.99–9.14)	4.69 (2.02–11.22)
**Asia (n = 97)^f^**						
Age	23	(19–27)	25	(21–29)	0.96 (0.89–1.02)	1.00 (0.93–1.06)
*S. stercoralis*	12/38	31.6	17/59	28.8	1.14 (0.46–2.75)	0.76 (0.25–2.13)
Hookworm^e^	10/31	32.3	3/48	6.3	7.14 (1.95–34.38)	7.54 (1.95–38.10)

CI: confidence interval; IQR: interquartile range; OR: odds ratio.

^a^ Infections were diagnosed using any of the following: urine microscopy or stool microscopy or serology.

**^b^** Adjusted for age and for the presence of other helminth infections through a multivariate logistic regression model.

**^c^** The total includes seven individuals coming from North Africa.

^d ^The value does not include one person from the cohort (n = 462) for whom info about eosinophil count is missing.

^e^ Includes *Ancylostoma duodenale* and *Necator americanus*.

^f^ No *Schistosoma* spp. was detected in individuals from Asia so it was not possible to adjust the model for this infection.

## Discussion

Infectious diseases are not the main problem affecting individuals that are part of the large, current wave of migration to Italy, often through the perilous Central Mediterranean route. Those who survive the journey almost invariably have, at least in our experience with a large number of asylum seekers, a history of experiencing physical, sexual and/or psychological harassment, violence and often torture. A recently published review underlines the role of traumatic experiences during the migration process on various aspects of health and health conditions [21]. Mental and psychosocial diseases, including depression and anxiety disorder, as well as the consequences of physical traumatism, are probably the first health priority to be dealt with [22,23].

However, attention should also be paid to infectious diseases, especially because of concerns about infection spreading to the local population that are often unfounded. In Italy therefore, the only screening formally indicated so far includes a clinical assessment for scabies and one for clinical and/or latent TB (protocols for the latter are not uniform). Many individuals are also screened for HIV, although there is no formal country-wide protocol for this infection, and a number are also screened for hepatitis B and C, with different, informal protocols.

At the CTD we applied, as a pilot initiative within the Veneto Region, an extended screening in Verona province that included screening for neglected helminthic infections. These are not a cause of concern for the local population, but may place a heavy clinical burden, even after many years, on infected individuals, including life-threatening complications of strongyloidiasis or schistosomiasis. The prevalence of neglected parasitic infections was high. These are most often asymptomatic, and therefore may only be detected with a specially designed screening, and they are never considered a priority as they cause no harm to the autochthonous population.

As far as HIV/AIDS is concerned, the prevalence in individuals from sub-Saharan Africa is low compared to the helminthic infections, and no individual from North Africa or Asia was found to be infected. The same is even more true for HCV infection as there was only one infected individual in the whole study population. On the contrary, prevalence of HBV infection was high and similar to that found by studies on other migrant populations [13,24]. Latent TB was high in our study population, which is similar to findings reported in recent papers from other European countries [13,24]. Is an extended screening such as the one we carried out worthwhile? A definitive answer would need to be based on a formal cost-effectiveness study, and that was well beyond the scope of this paper. However, we believe that our results certainly question the usefulness of screening for HCV infection because of its low prevalence in combination with extremely high-cost treatment that has limited its use to advanced stages of the disease. The utility of HIV screening is also debatable, as is that of HBV infection. Both imply treatments that, besides being very expensive, require a long-term management and a treatment compliance that is particularly difficult to obtain in this often very mobile population. The same problem, though to a lesser extent, concerns latent TB screening: shorter courses, i.e. 3 months, of treatment are available and some local health units in our region have been able to achieve 80% or more of treatment success (personal communication, C. Postiglione April 2018). However, others simply refrain from providing any treatment as they feel unable to ensure sufficient compliance and follow-up in this group of individuals. We argue that if treatment is not offered and adequate conditions to guarantee compliance cannot be ensured through directly observed treatment (DOT) or a similar strategy, latent TB screening is not a good use of resources and should not be proposed.

Helminthic infections can also potentially cause serious health consequences for infected individuals, however, person-to-person transmission does not represent an issue and treatment is comparatively straight forward. Like our study, other studies reported high prevalence of some helminths targeted by the screening of asymptomatic individuals [14,25]. While the implementation of screening activities for these infections sometimes faces obstacles, such as refusal to supply biological samples and test limitations (low sensitivity of stool examination in case of *Schistosoma* spp. and *S. stercoralis* infections, and concerns about specificity of serology), treatment of these infections is short, often a stat dose, very effective, well tolerated and reasonably cheap [14]. Two studies of cost-effectiveness of the management of this group of diseases in immigrants were carried out in the United States (US) [17,26], with a particular focus on strongyloidiasis, concluding that presumptive treatment was the more cost-effective option, especially if provided in the country of origin before departure.

These studies from the US are not particularly applicable to the Italian situation for several reasons. First, schistosomiasis was highly prevalent in our study, but, reflecting a different geographical origin of immigrants, the American studies did not consider this infection in their analyses. Second, treatment before migration is obviously not possible given that many migrants are arriving in Europe without having previous contact with authorities. Third, drug availability and cost are a major concern in Italy given that ivermectin and praziquantel, which are used for strongyloidiasis and schistosomiasis, respectively, are not registered and need to be imported at a non-negligible cost. Fourth, by the Italian Constitution, every individual in Italy has the right to the best-available healthcare which means that any difference in medical approach between Italians and non-Italians would be discriminatory. Although offering screening for helminthic infections only to symptomatic people might presumably increase the compliance to diagnostic tests and treatment, this approach would considerably reduce asymptomatic infected individuals access to treatment. Also, as schistosomiasis and strongyloidiasis frequently lead to chronic indolent diseases, such a strategy would leave the largest proportion of infected individuals, asymptomatic individuals, at high risk of potentially-fatal complications. A halfway measure, which also takes difficulties in obtaining the stool samples into consideration, might be presumptive treatment for helminthic diseases based on the presence of eosinophilia. This might be a particularly valid option for population subgroups and/or helminths where the association between helminths and eosinophilia proved to be strong. In comparison with a strategy that screens symptomatic people, a smaller proportion of infected individuals would be left without treatment. Moreover, this approach would reduce the costs and the logistical constraints of universal screening. However, the negative predictive value of eosinophilia might not be sufficiently high to safely exclude strongyloidiasis and schistosomiasis, as suggested previously [14]. An in-depth cost-effectiveness analysis should be conducted.

In any case, the current strategy of not addressing neglected parasitic diseases is not acceptable.

A main limitation of this study is that the cohort of asylum seekers was almost entirely composed of males so we could not evaluate possible, sex-related differences in the distribution and proportion of the same infections. Moreover, our results may not be representative of the situation of asylum seekers in other countries in Europe/other Italian settings. The main strength is that this paper adds to the little data available in the medical literature on an extensive screening of newly-arrived asylum seekers that includes helminthic infections, regardless of whether or not clinical symptoms are present. We believe that our data on a cohort of individuals, mostly originating from sub-Saharan Africa, particularly contribute to filling a gap of knowledge in terms of relevant helminthic infections possibly presented by asylum seekers in Italy. This may prove particularly useful as the Italian Ministry of Health recently issued new screening guidelines that recommend the screening of asylum seekers and refugees for strongyloidiasis and schistosomiasis for the first time [27].
